# Molecular Heterogeneity of High Grade Colorectal Adenocarcinoma

**DOI:** 10.3390/cancers13020233

**Published:** 2021-01-10

**Authors:** Cristian Perna, Antonia Navarro, Ignacio Ruz-Caracuel, Tamara Caniego-Casas, Eva Cristóbal, Susanna Leskelä, Federico Longo, Alejandra Caminoa, Almudena Santón, Reyes Ferreiro, David Pizarro, María Luisa Palacios-Berraquero, José Palacios

**Affiliations:** 1 Department of Pathology, Hospital Universitario Ramón y Cajal, Instituto Ramón y Cajal de Investigación Sanitaria, 28034 Madrid, Spain; luiscristian.perna@salud.madrid.org (C.P.); antonia.navarro@salud.madrid.org (A.N.); ignacio.ruz@salud.madrid.org (I.R.-C.); tamara.caniego@salud.madrid.org (T.C.-C.); evamaria.cristobal@salud.madrid.org (E.C.); susanna.leskela@salud.madrid.org (S.L.); mariaalejandra.caminoa-lizarralde@salud.madrid.org (A.C.); almudena.santon@salud.madrid.org (A.S.); david.pizarro@salud.madrid.org (D.P.); 2 Departamento de Medicina y Especialidades Médicas, Facultad de Medicina, Universidad de Alcalá, 28029 Madrid, Spain; 3 CIBERONC, Instituto de Salud Carlos III, 28029 Madrid, Spain; federico.longo@salud.madrid.org (F.L.); mariareyes.ferreiro@salud.madrid.org (R.F.); 4 Department of Medical Oncology, Hospital Universitario Ramón y Cajal, 28034 Madrid, Spain; 5 Department of Hematology and Hemotherapy, Clínica Universidad de Navarra, 31008 Pamplona, Spain; mpalaciosb@unav.es

**Keywords:** colorectal cancer, next generation sequencing, mismatch repair, microsatellite instability, gastrointestinal pathology

## Abstract

**Simple Summary:**

Due to its low frequency, high grade colorectal carcinomas (HG-CRCs) are underrepresented in molecular series. We intended to further characterize the pathological and molecular features of these tumors. In addition, morphologically different areas when present, were analyzed separately to study tumor heterogeneity. We found that most (72.5%) of HG-CRCs showed mismatch repair (MMR) deficiency. MMR status conditioned the frequency and the clonality of the molecular alterations found. Thus, whereas *BRAF* mutations and gene fusions were observed only in MMR deficient (MMRd) tumors, *TP53*, *KRAS*, and gene amplifications predominated in MMR proficient (MMRp) tumors. In MMRp tumors, gene amplification was a mechanism of progression, whereas the accumulation of mutations in genes of different pathways such as *NOTCH*, MMR or *PIK3CA* was involved in the clonal diversity of MMRd HG-CRC. In summary, intertumor and intratumor molecular heterogeneity in HG-CRCs is mainly due to MMR status.

**Abstract:**

High grade colorectal carcinomas (HG-CRCs), which comprise 15% of colorectal carcinomas, are underrepresented in reported molecular studies. Clinicopathological, immunohistochemical, and molecular features of 40 HG-CRCs are described. Moreover, glandular and solid areas of 25 tumors were separately analyzed. The expression of MLH1, PMS2, MSH2, MSH6, p53, E-cadherin, CDX2, CK20, CD8, PDL1, PAN-TRK, c-MET, SMARCB1, ARID1A, SMARCA2, and SMARCA4 was analyzed by immunohistochemistry. Promoter *MLH1* methylation was analyzed in tumors with MLH1/PMS2 loss. Next-generation sequencing was used to screen 161 genes for hotspot mutations, copy number variations and gene fusions. In this series, 72.5% of HG-CRCs showed mismatch repair deficiency (MMRd). MMR deficient tumor and MMR proficient (MMRp) tumors showed striking molecular differences. Thus, whereas BRAF mutations were only observed in MMRd tumors, mutations in *KRAS* and *TP53* were more frequent in MMR proficient tumors. Moreover, gene fusions (*NTRK1* and *MET*) were detected only in MMRd tumors, whereas gene amplification (*MYC*, *CCND1* and *EGFR*) predominated in MMRp/*TP53*-mutated tumors. Loss of expression of proteins involved in chromatin remodeling, such as ARID1A, was observed only in MMRd HG-CRCs, which also showed more frequently PD-L1 expression and a higher number of tumor infiltrating lymphocytes. The separate analysis of glandular and solid areas indicated that the clonal or subclonal nature of the molecular alterations also depended on MMR status. Mutations in genes such as *TP53* and *KRAS* were always clonal in MMRp-CRCs but occurred as subclonal events in MMRd-CRCs. Gene amplification was implicated in the progression of MMRp tumors, but not in MMRd tumors, in which clonal diversity was due to accumulation of mutations in genes of different pathways such as *NOTCH*, MMR, or *PIK3CA*. In summary, intertumor and intratumor molecular heterogeneity in HG-CRCs is mainly due to MMR status.

## 1. Introduction

Histological grade is not only an important prognostic factor in colorectal carcinoma (CRC), but is also a variable used for therapy selection. Grading is performed on the basis of architectural criteria. Recently, the new World Health Organization (WHO) classification of gastrointestinal tumors has recommended the denomination of high-grade CRC (HG-CRC) to those previously called poorly differentiated (PD) CRCs, which are characterized by <50% of glandular differentiation [1]. It is estimated that around 15% of CRC are PD, however reported frequencies ranged from 2.5% to 30% [2,3,4], indicating only fair interobserver reproducibility [4]. HG-CRCs do not only include conventional CRC with a predominant solid component, but also some special histological types characterized by a solid growth pattern, such as medullary carcinoma (MC) and undifferentiated carcinoma (UC). According to WHO’s definition, MC is characterized by sheets of malignant cells with vesicular nuclei, prominent nucleoli, and abundant eosinophilic cytoplasm. In addition, these tumors have a prominent infiltration by lymphocytes and neutrophils and pushing borders. UC lack morphological, immunohistochemical, and molecular evidence of differentiation beyond an epithelial tumor. Moreover, they differ from medullary carcinoma in their lack of pushing borders, syncytial growth pattern, and prominent lymphoplasmacytic infiltration [1].

However, the diagnosis of MC and or UC based on histological criteria is difficult and suffers of lack of reproducibility. Thus, two studies have reported that diagnostic reproducibility of MC was modest with a ĸ value of only 0.32 [5,6]. In addition, some authors consider the presence of mismatch repair deficiency (MMRd) necessary for the diagnosis of MC, whereas others note a very strong association but accept that some medullary MC may not show MMRd. For example, in the study of Gonzalez et al [7], the authors studied colonic carcinomas with a solid pattern and considered MCs those showing MMRd and UCs those that were MMR proficient (MMRp). Interestingly, the authors only observed statistically significant differences between both tumors’ groups in lymphocyte and neutrophil infiltration, but no differences were observed regarding syncytial growth, pushing margins, nuclear pleomorphism, prominent nuclei, necrosis, and mitotic activity, among others.

During the last years, several studies have performed a comprehensive molecular characterization of CRC [8]. However, to the best of our knowledge, a detailed analysis of molecular alterations based on the histological grade has not been performed. Due to low frequency of HG-CRCs, this group of tumors is usually underrepresented in most reported series.

Most studies analyzing intratumor heterogeneity (ITH) in CRC have evaluated temporo-spatial differences between primary tumors and metastasis [8]. Only few studies have evaluated spatial ITH in primary tumors by analyzing different areas of the tumor [9,10], the allelic frequency of the mutations [11,12,13], or by single cell analysis [8]. However, to the best of our knowledge, the possible molecular differences between the glandular and solid areas of CRCs have not been analyzed previously.

In this study we analyzed the clinical, pathological, immunohistochemical and molecular features of a group of 40 HG-CRCs. In addition, we analyzed immunohistochemical and molecular features of glandular and solid areas in tumors with both components, to evaluate ITH and gain insight into the molecular mechanism involved in tumor progression.

## 2. Results

### 2.1. Mismatch Repair Status

A total of 40 CRCs with >50% of solid component were retrieved. For clarity, and because MMR status was related with clinical, pathological, and molecular features, we first presented MMR results. A total of 29 CRCs (72.5%) showed loss of expression of at least 1 MMR protein (Table S1): MLH1/PMS2 in 21 tumors, MSH2/MSH6 in 3 tumors, MLH1/PMS2/MSH6 in two tumors, PMS2 in 1 tumor, MSH6 in one tumor, and MLH1/PMS2/MSH2/MSH6 in 1 tumor.

In 27 tumors, we demonstrated at least one molecular alteration in MMR genes. The most frequent alteration was *MLH1* promoter hypermethylation, which was present in 21 out of 24 tumors with loss of at least MLH1/PMS2. Two additional cases had an invalid result.

We detected *MSH2* pathogenic mutations in two tumors with MSH2/MSH6 loss and a pathogenic *PMS2* mutation in a tumor with isolated PMS2 loss of expression (Table S1). According to allelic frequencies, and the lack of these mutations in normal tissue, we considered them somatic mutations. However, since we did not perform additional techniques such as Multiplex ligation-dependent probe amplification (MLPA) to detect large deletions, we could not exclude Lynch syndrome in these patients.

We were able to demonstrate Lynch syndrome in only one out of 29 (3.4%) patients with MMRd tumors. This patient carried a germline mutation in *MSH6* (T29), which was also present in non-tumor tissue. In a tumor showing the absence of expression of MSH2/MSH6, we did not find any molecular alteration in MMR genes (T27).

### 2.2. Clinicopathological Features

The main clinicopathological features and differences according to MMR status are presented in Table 1. Clinicopathological data of each patient are presented in Table S2.

Mean age of MMRd CRCs was 69.79 years compared to 74.64 years in MMRp CRCs. Regarding location, 75.9% and 72.7% of MMRd and MMRp CRCs respectively affected the right or transverse colon. All MMRp CRCs presented in stage III or IV, whereas 31.0% of MMRd CRCs presented in stages I and II.

In 25 cases (62.5%), areas of glandular differentiation were present. Focal (<10%) mucinous differentiation was present in four cases (three MMRd and one MMRp). MMR status is key to grade and to describe clinicopathological features of CRC.

### 2.3. Molecular Alterations in High Grade Colorectal Carcinoma

**Figure 1:**
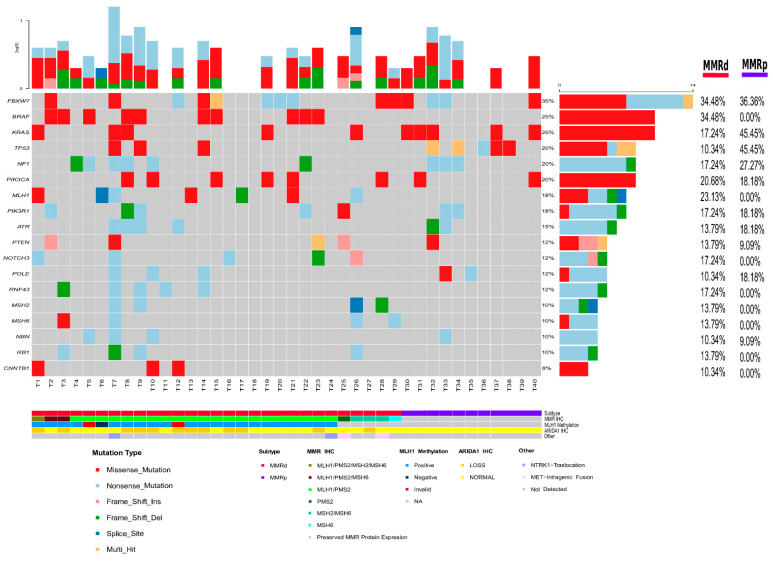
Summary of the molecular characteristics observed in high-grade colorectal carcinomas.

The complete set of mutations detected in glandular and solid areas in this study is presented in Tables S1 and S3. Next generation sequencing average coverage was above 85% and the average depth was above 800X. The most frequent mutations detected in this series are presented in Figure 1. This Figure also shows the mutation frequencies according to MMR status. The most frequent mutated genes in the complete series were *FBXW7*, *BRAF*, *KRAS*, *TP53*, *NF1,* and *PIK3CA*. *BRAF* mutation were more frequent in MMRd tumors. In contrast, *KRAS* and *TP53* were more frequently mutated in MMRp. Mutations that were overrepresented in MMRd included, among others, those affecting *BRAF*, *MLH1*, *NOTCH3*, *RNF43*, and *CTNNB1*. Mutations in *PIK3CA*, *PIK3R1*, and *PTEN*, three components of the PIK3 pathway, were mutated at a similar frequency in MMRd and MMRp tumors.

Only one MMRp tumor carried a pathogenic mutation (P286C) involving the exonuclease domain of *POLE* (T33).

For comparison, CRCs data from Giannakis et al [14] cohort was analyzed (Figures S1–S4 and Table S6). The series included 47 HG-CRCs among 619 CRCs reported. HG-CRCs included 25 (53.19%) MMRd tumors, and 20 (42.55%) MMRp tumors. MMR status was not reported in 2 tumors. When we analyzed the same 114 genes contained in our panel, the most frequent mutated genes in Giannakis´series [14] were: *BRAF*, *TP53*, *RNF43*, *PIK3CA*, *FBXW7*, *ARID1A*, and *ATM*. *BRAF* was the most frequent mutated gene among MSI tumors and *TP53* among MSS tumors. In contrast to our results *KRAS* mutations were in lower frequencies. We also analyzed in Giannakis´series [14] molecular differences according to histological grade in both MMRd and MMRp tumors. The main differences are presented in Figures S3 and S4. Interestingly, *BRAF* and *RNF43*, two genes frequently mutated in MMRd CRCs, were also more frequently mutated in MMRp HG-CRCs than in LG-CRCs in Ginnaki’s series. These results could be in line with those recently reported by Wang et al [15] who observed that *APC* wild type MMRp CRCs had poor prognosis and that in these tumors *BRAF* and *RNF43* mutations were overrepresented. Unfortunately, histological grade was not reported in Wang’s study.

In our series, we found gene amplifications in 4 tumors. Among MMRp CRCs, we found *MYC* and *FLT3* amplification in one tumor (T32), *MYC* and *CCND1* amplification in 1 tumor (T36), and *EGFR* amplification in one tumor (T38). Three of these tumors carried *TP53* mutations. Only one MMRd CRC had *MYC* amplification (T26).

**Figure 2:**
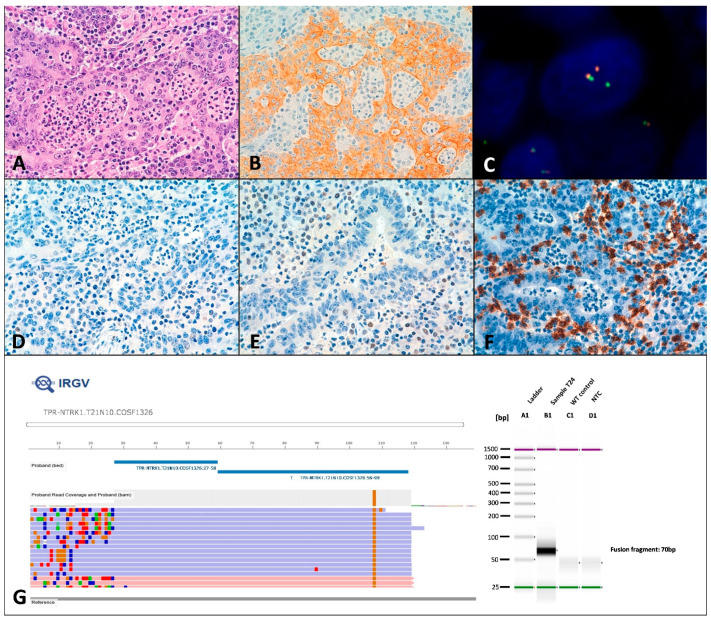
*NTRK* fusion in tumor T7. (**A**) Hematoxylin–eosin staining showing a solid carcinoma with a lymphoid infiltrate. (**B**) PAN-NTRK immunohistochemistry had a diffuse positive membrane patter. (**C**) ZytoLight SPEC NTRK1 FISH showing separate signals. (**D**) MLH1 and (**E**) PMS2 loss of expression. (**F**) CD8 highlights the abundant lymphoid infiltrate. (**G**) On the left, Ion Reporter Genomic Viewer (IRGV) image, corresponding to the *TPR(21)-NTRK1(10)* fusion. On the right, fusion validation by RT-PCR.

A total of 3 intergenic gene fusions were found. Two intergenic gene fusions were detected by Next-generation sequencing (NGS). One MMRd tumor carried the intergenic fusion *TPR(21)-NTRK1(10)*, which was additionally validated by RT-PCR, Sanger sequencing, immunohistochemistry and FISH (T7) (Figure 2). Moreover, another tumor carried the intergenic fusion *TBL1XR1(1)–PIK3CA(2)* (T1), although orthogonal validation using RT-PCR and Sanger sequencing could not confirm its presence. In addition, one *NTRK1* fusion was detected by FISH in a tumor overexpressing panNTRK (T24). No fusion was detected in this case by NGS, despite multiple areas of the tumor were analyzed. Moreover, the intragenic fusion *MET(13)-MET(15)* (MET exon 14 skipping) was detected in two tumors (T25 and T28) and validated by RT-PCR and Sanger sequencing.

### 2.4. Molecular Differences between Glandular and Solid Areas

In order to find molecular alterations related to tumor progression, we analyzed both glandular and solid areas in 25 tumors showing two morphological differentiated areas (Table S3 and Figure S5). All but one tumor shared at least one molecular alteration in both components, indicating clonal relationships. The only tumor (T31) in which different mutations were observed between both areas was a MMRp tumor showing a *KRAS* mutation in the glandular area and *PIK3CA* mutation in the solid area. However, we cannot exclude a clonal relationship of both components since our panel did not include *APC*, which is the most frequent mutated gene in MMRp CRC.

**Figure 3:**
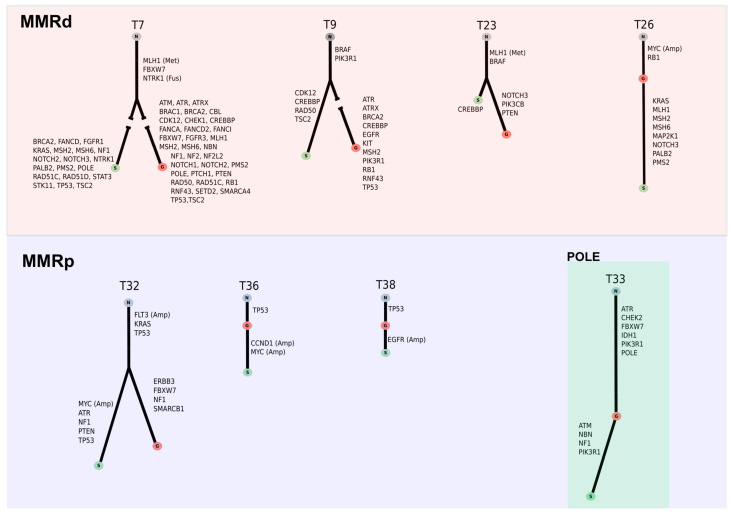
Evolutionary trees of different high-grade colorectal carcinomas that had both solid and glandular areas. The length of the branches is proportional to the number of mutated genes in each sample.

In four MMRd CRCs and two MMRp CRCs (including the only case with POLE mutation) we observed three or more different molecular alterations between the glandular and solid areas (Figure 3). In three MMRp tumors with *TP53* mutations, amplification of *EGFR* (T38), *CCND1* (T36), and *MYC* (T32 and T36) were detected in the solid component but not in the glandular areas, which were validated by FISH.

*KRAS* and *TP53* mutations, each of which were present in 3 MMRd tumors, occurred as subclonal events in two tumors, respectively. In contrast, in MMRp CRCs *TP53* and *KRAS* were always present in the two analyzed areas suggesting the clonal nature of the mutation. *FBXW7* and *PIK3CA* mutations, which were frequently mutated in both MMRd and MMRp tumors, were always clonal in MMRd, but were subclonal in three and two MMRp tumors, respectively.

### 2.5. Immunohistochemical Features

**Figure 4:**
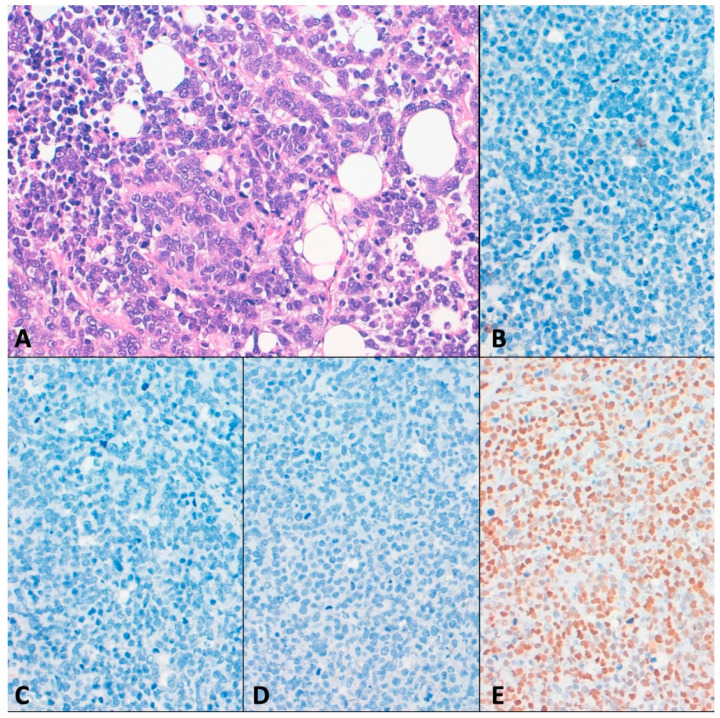
MMR proficient tumor 30 (**A**) Hematoxylin–eosin staining showing a solid tumor. There was absence of expression of (**B**) CDX2 and (**C**) CK20. In addition, (**D**) E-cadherin expression was lost. (**E**) p53 had an aberrant pattern without any mutation in TP53 discovered by NGS analysis.

To further characterize HG-CRCs in this series, several immunohistochemical markers were analyzed (representative figures of positive and negative tumors can be found in Figure S6). Among differentiation markers, CK20 was positive in nine (81.8%) MMRp CRCs and in 16 (55.2%) MMRd CRCs. Moreover, CDX2 was retained in seven (63.6%) MMRp and in 14 (48.3%) MMRd. Therefore, loss of expression of CK20 and/or CDX2 was more frequent in MMRd CRCs than in MMRp CRCs (Figure 4A–C), although these differences were not statistically significant (Table 1). E-cadherin was completely lost in only one tumor that was MMRp (T30) (Figure 4D). Additionally, three tumors (two MMRd and one MMRp) showed E-cadherin loss in solid-undifferentiated areas (T9, T29, and T40).

P53 immunohistochemistry exhibited an aberrant positive pattern in six tumors (five MMRp and one MMRd) (Table 1), that correlate with *TP53* mutation in five cases. One MMRp tumor showing an aberrant p53 pattern had any mutation discovered in *TP53* in the genomic analysis (Figure 4E). Three additional cases having mutations in *TP53* (T7, T9, and T36) exhibited native p53 immunohistochemical patterns.

**Figure 5:**
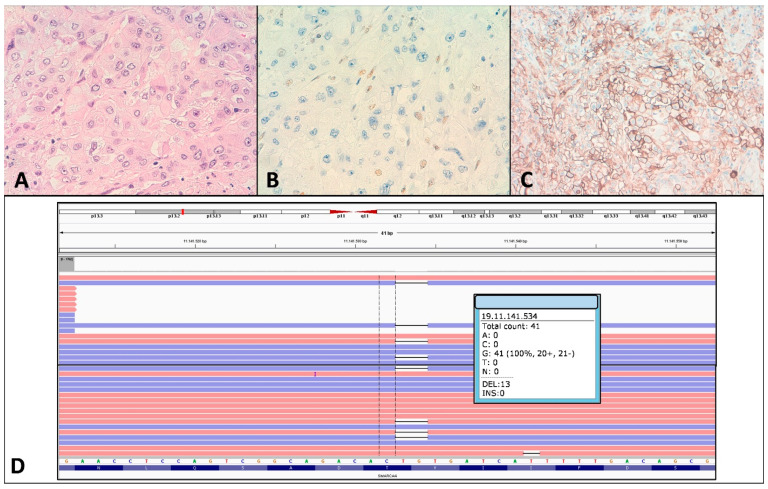
Tumor T19 mutated in *SMARCA4*. (**A**) Hematoxylin-eosin showing a high-grade colorectal carcinoma. (**B**) SMARCA4 immunostaining showing a complete loss of expression. (**C**) PDL1 had a membranous positivity in more than 50% of epithelial cells. (**D**) IGV view of *SMARCA4* c.3512_3513delTG (p.Val1171fs) mutation.

We analyzed the expression of several chromatin-remodeling factors. ARID1A loss was observed in 12 cases, including two cases with focal loss affecting only solid areas of the tumor. ARID1A loss of expression only occurred in MMRd CRCs (Table 1). In addition, we observed one tumor with complete absence of SMARCA4 expression (T19) (Figure 5A,B) which was associated with a truncation mutation (p.Val1171fs) in *SMARCA4* (Figure 5D) and one additional tumor with focal loss in areas of dedifferentiation (T10). No alterations in the expression of INI1 (SMARCB1) and SMARCB2 were detected.

**Figure 6:**
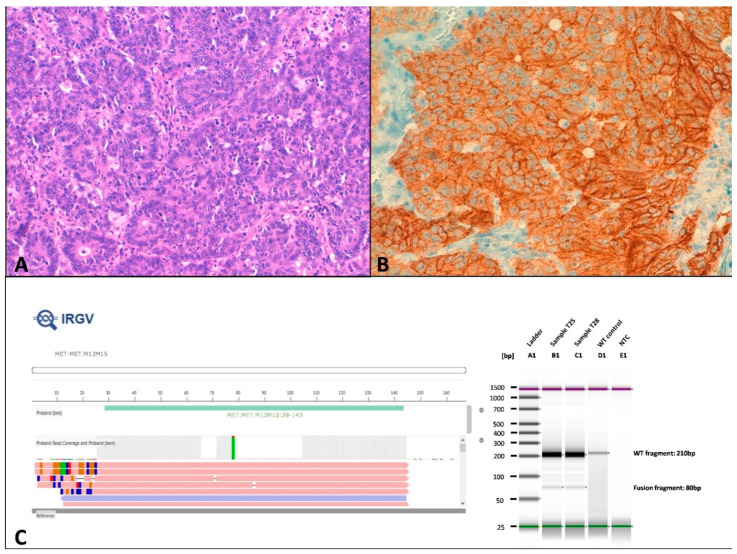
c-*MET* traslocation in tumor T28. (**A**) Hematoxylin-eosin showing a grade 3 tumor with scarce glandular formation. (**B**) c-MET immunostaining was diffuse and intense. (**C**) On the left, Ion Reporter Genomic Viewer (IRGV) image, corresponding to the *MET(13)-MET(15)* fusion. On the right, fusion validation by RT-PCR.

C-MET was overexpressed in 16 tumors and it was significantly overexpressed in a higher proportion of MMRd tumors (55.2%) compared with MMRp tumors (9.1%) (*p* = 0.008) (Table 1 and Figure 6A,B). Both tumors showing the intragenic fusion *MET(13)-MET(15)* (*MET* exon 14 skipping) where associated with positive c-MET staining (T25 and T28) (Figure 6C). PanNTRK immunohistochemistry showed positive membrane staining in two tumors (T7 and T24) that harbored *NTRK1* intergenic fusion detected by FISH (Figure 2A–C).

PD-L1 expression in over 1% of tumor cells was observed in 17 (58.6%) MMRd CRCs cases (Figure 5C) and in two (18.2%) MMRp. PD-L1 was expressed in more than 50% of tumor cells in only 2 MMRd CRCs (T3 and T19) (Figure 5C). The mean number of intraepithelial lymphocytes (TILs) per HPF was 10.38 for MMRd CRCs and 2.27 for MMRp. 26 MMRd tumors (89.7%) (Figure 2F) and 1 MMRp case (9.1%) showed three or more TILs per HPF.

### 2.6. Survival

**Figure 7:**
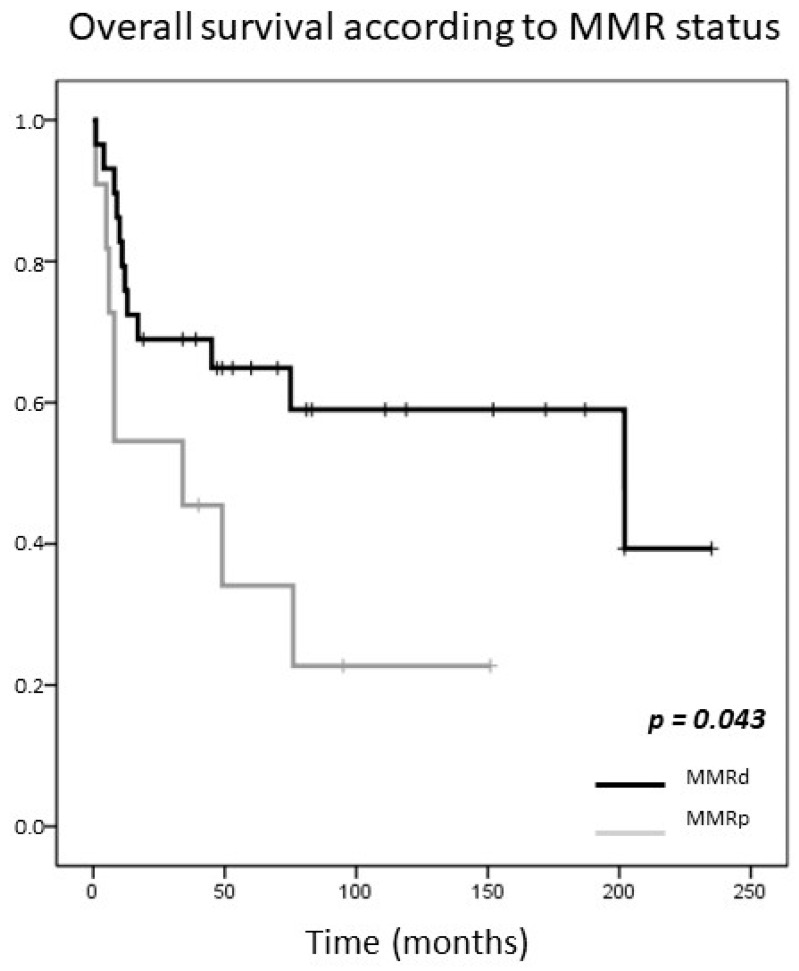
Kaplan-Meier curve for overall survival according to the mismatch repair status.

After a mean follow-up of 65 months, there were 12 deaths (41.4%) among patients with MMRd CRCs and eight (72.7%) deaths among patients with MMRe CRCs. In the univariate analysis MMRd CRCs showed better survival (log rank *p* = 0.043, HR = 0.40, 95% CI = 0.16–1.01, *p* = 0.052) (Figure 7). Overall survival was also associated with higher stage (log rank *p* = 0.024, HR = 4.74, 95% CI = 1.07–20.95, *p* = 0.04). Thus, in the multivariable analysis, after adjusting for stage, the differences in survival were not statistically significant (HR = 0.58, 95% CI = 0.23–1.49, *p* = 0.257).

## 3. Discussion

In this study, we analyzed the clinical, pathological, inmunohistochemical and molecular features of a group of HG-CRCs and demonstrated intertumor heterogeneity due to MMR status, as well as intratumor heterogeneity between areas with glandular and solid growth pattern.

From a morphological point of view, we did not try to differentiate among conventional adenocarcinoma, MC and UC due to lack of reproducibility of morphological criteria for the diagnosis of MC and the lack of objective biomarkers for the diagnosis of UC.

The frequency of MMRd in our series of HG-CRCs was 72.5%, a proportion similar to the 78% reported by Gonzalez et al [7] in a series of 51 HG tumors, and slightly higher than the 53.19% reported by Giannakis et al. [14] in a series of 47 HG tumors.

There are few NGS studies analyzing molecular differences in CRCs according to MMR status [14,16,17,18,19] and there is specific data on HG-CRCs in only one of them [14]. Our series, as well as previous studies, shows that MMRd CRCs had a different mutational profile when compared to MMRp CRCs, and demonstrated that the most common mutated gene in MMRd CRCs was *BRAF*, whereas in MMRp CRCs were *KRAS* and *TP53*. The high proportion of *BRAF* mutations in MMRd CRCs is associated with the high prevalence of tumors with *MLH1* promoter hypermethylation. We did not observe *BRAF* mutations in MMRp CRCs. In contrast, *KRAS* occurred in both groups of tumors. However, *BRAF* and *KRAS* mutations were mutually exclusive, since only 1 out of 14 MMRd CRCs that carried mutations in *BRAF* and/or *KRAS* genes had mutations in both genes.

*FBXW7*, a component of the *NOTCH* pathway, was frequently mutated in both MMRd and MMRp CRCs. In addition, *FBXW7* mutations were mutually exclusive with *TP53* mutations in MMRp CRCs, since only one out of eight tumors that carried *TP53* and/or *FBXW7* had both mutations. A previous study has recently reported that MMRd CRC are enriched in mutations affecting the *NOTCH* pathway. In this line, we only observed mutations in *NOTCH1*, *NOTCH2* and *NOTCH3* in MMRd tumors. In accordance with results reported by Trabucco et al [18] we also observed an increased frequency of mutations in the MMR genes *MLH1*, *MSH2* and *MSH6*, and in the *WNT* genes (*RNF43* and *CTNNB1* -beta-catenin-) in MMRd.

Recent studies have demonstrated that a low percentage of CRCs carry oncogenic fusions (OF), which confer specific clinicopathologic features to the tumors. Recent studies have reported that OF are more prevalent among sporadic MMRd CRCs due to *MLH1* promoter hypermethylation, that are *KRAS* and *BRAF* wild type [20,21]. Thus, whereas the frequency of OF is <1% in the unselected population of CRCs, this frequency rises to 5% in MMRd CRCs and to more than 50% in MMRd/*KRASwt*/*BRAFwt* CRCs [20,21,22,23]. In our series, we detected by NGS and/or FISH 2 intergenic fusions affecting *NTRK1* (with panTRK overexpression) and 2 MET intragenic fusions, which were found in MMRd CRCs with wild type *KRAS* and *BRAF*. However, whereas *NTRK1* fusions occurred in tumors with loss of MLH1 due to *MLH1* promoter hypermethylation, the two *MET* intragenic fusions occurred in CRCs with somatic mutations in *MSH2* and *PMS2*, respectively. Only a previous series have reported *MET* fusions in four CRC [24]. In both series, *MET* fusions were associated with MET overexpression. In addition, 15 cases in present series overexpressed MET without *MET* fusion or amplification. MET overexpression by immunohistochemistry has been reported in 33% to 72% of CRC and has been correlated with worse overall and disease-free survival [25,26]. Interestingly, in our series there was only overexpression in 1 MMRp case, in contrast to previous reports, describing 73% vs 59% in MMRp and MMRd cases respectively [26].

Consistent with the role of *TP53* in promoting chromosome instability, we observed that gene amplification was more frequent in MMRp (27%) than in MMRd (9%) CRCs. Although the sample of MMRp CRCs is small, we observed a relatively higher frequency of amplification in *MYC* (18%), *EGFR* (9%) and *CCND1* (9%), than previously reported in largest CRC series, which reported 4.8% to 5.1% of *MYC* amplification, 0.7% to 1.4% of *EGFR* amplification and 0.2% to 0.7% for *CCND1* amplification [19,27]. These data, together with the observation that amplifications occurred as subclonal events in solid areas, suggest that gene amplification is associated not only with high grade, but also with progression in CRC.

Considering differentiation markers, we observed several immunohistochemical differences between MMRd and MMRp tumors. In our series CK20 was positive in 55.2% and 81.8% of MMRd and MMRp tumors, respectively. Similar results have been previously described considering all grades, 37% in MMRd and 88% in MMRp cases [28]. Discriminating by grade, a 46% and 81% of CK20 positivity has been reported in high-grade and low-grade CRC, respectively [29]. In our results, CDX2 was positive in 48.3% of MMRd cases and in 63.6% of MMRp cases, according with previous results considering all grades [30]. However, some authors have reported more elevated rates of positivity in both MMRd and MMRp tumors, probably related to the clone used [31].

In present series, only a MMRp CRCs that lacked evidence of differentiation and did not express CK20 and CDX2 showed complete loss of E-cadherin. In contrast, in endometrial cancer, a tumor with high frequency of MMRd, we have previously reported that lack of E-cadherin expression is a good subrogate for the definition of undifferentiated carcinoma [32]. In addition, 2 MMRd and 1 MMRp tumors lost E-cadherin focally in solid/undifferentiated areas, with retention of E-cadherin in more differentiated areas, a situation reminiscent to that reported in dedifferentiated endometrial carcinoma, a histological type that occurs more frequently in the context of MMRd [33].

One of the most striking differences that we observed between MMRd and MMRp CRCs was the loss of expression of ARID1A in 41% of MMRd tumors. According to a recent metanalysis, the frequency of *ARID1A* mutation and ARID1A protein expression loss in CRC patients was approximately 12–13% and was significantly associated with poorly differentiated grade and advanced tumor depth [34].

In the present series, in addition to ARID1A we also evaluated the expression the SWI/SNF complex components SMARCB1 (INI1), SMARCA4 (BRG1) and SMARCA2. We only found one case with complete loss of SMARCA4. This tumor carried a somatic mutation of the gene, which probably was associated to a second hit not detected in our molecular analysis. In addition, focal loss of SMARCA4 was observed in undifferentiated areas of one additional tumor, with retained expression in the glandular areas. Absence of SMARCA4, which is typical of the small cell carcinomas of the ovary [35] and occurs in some undifferentiated/dedifferentiated endometrial carcinomas [36], seems to be very infrequent in CRC. To the best of our knowledge, no cases of complete absence of expression have been reported. Vanacker et al [37] reported a case in which a *SMARCA4* mutation was detected in the neuroendocrine component of a mixed adenoneuroendocrine carcinoma of the colon, but no immunohistochemical study was performed to confirm its impact on protein expression. The loss of SMARCA4 expression has been reported occasionally in undifferentiated carcinomas with rhabdoid features in the small intestine and stomach [38,39].

PD-L1 expression and the presence of TILs has been associated with microsatellite instability in CRC [40,41]. In our series of high-grade CRCs, we confirmed this association, PDL1 was expressed in more than 1% of epithelial cells in 58.6% of MMRd tumors and in 18.2% of MMRp tumors. Median of TILs per HPF in MMRd cases was 10.38 in contrast with 2.27 in MMRp cases. This fact supports the use of immunotherapy in MMRd CRCs [42].

In this study, we analyzed glandular and solid areas in 25 CRCs and demonstrated the clonal relationship between them. Although in most cases the number of different alterations in both areas were limited, some cases showed a high degree of ITH, as illustrated by tumor T9 differing in 125 mutations between the two components. A higher degree of ITH was detected, as expected, in MMRd CRCs, and in the POLE-mutated tumor. In the three tumors with the higher degree of ITH, subclonal mutations in additional MMR genes occurred in the solid areas, suggesting a role of secondary MMR gene mutations in progression.

Studies on spatial heterogeneity in primary CRCs are scarce and have suggested that mutations in *APC*, *TP53*, *KRAS*, and *FBXW7* are clonal, whereas alterations in *PIK3CA* and *MYC* are frequently subclonal [9,12]. However, these studies did not differentiate between MMRd and MMRp CRCs. Our results suggested that the clonal or subclonal nature of the molecular alteration in one specific gene depends on the molecular context. Thus, genes whose mutations were always clonal in MMRp CRCs, such as *TP53* and *KRAS*, occurred as subclonal events in some MMRd CRCs. Moreover, genes which mutations were always clonal in MMRd CRCs, such as *FBXW7* and *PIK3CA*, occurred as subclonal events in some MMRp CRCs. In addition to these molecular differences, the most striking immunohistochemical differences between solid and glandular areas were the complete loss of SMARCA4 in the solid component in one tumor and the focal loss of ARID1A in the undifferentiated area of two tumors. Moreover, the loss of E-cadherin was proven in solid areas of three tumors that retained its expression in the glandular component. All but one of these focal losses of E-cadherin occurred in MMRd.

MMRd is reported to be a favorable prognostic factor in CRC [16]. Accordingly, we observed a longer survival in MMRd HG-CRCs in the univariate analysis. However, no significant differences in survival were found between MMRd and MMRp HG-CRCs after adjusting for stage, although this should be interpreted with caution due to the limited number of cases included in the study.

## 4. Materials and Methods

### 4.1. Cohort Selection

Tumors with a diagnosis of poorly differentiated and undifferentiated CRC in our institution were retrieved and reviewed by three Pathologists (CP, AN, JP). Final selection criteria were a solid pattern >50% and tissue available for molecular and immunohistochemical analysis. Cases with mucinous differentiation were excluded except if it was less than 10% of the tumor. Clinical (age, localization treatment, and outcome) and pathological data (size, lymph node metastasis) were obtained from clinical and pathological records.

### 4.2. Immunohistochemistry

Representative areas of the tumors were selected on hematoxylin and eosin-stained sections and marked on individual paraffin blocks. Two tissue cores (1 mm in diameter) were obtained from each specimen. The tissue cores were arrayed into a receptor paraffin block using a tissue microarray (TMA) workstation (Beecher Instruments, Silver Spring, MD, USA). Cores from the solid and glandular areas were obtained (when available). Immunohistochemistry was performed on TMA sections using the following antibodies: MLH1, PMS2, MSH2, MSH6, p53, E-cadherin, CDX2, CK20, CD8, PDL1, PAN-TRK, c-MET, SMARCB1, ARID1A, SMARCA2, and SMARCA4. Clones, suppliers, and dilutions are presented in Table S4.

### 4.3. Nucleic Acid Extraction and Sequencing

For nucleic acid extraction, paraffin blocks were punched in the area selected in the hematoxylin and eosin-stained tissue sections containing at least 75% of tumor cells. DNA and RNA were extracted using RecoverAll Total Nucleic Acid Isolation Kit for FFPE (Invitrogen, Carlsbad, CA, USA) following the manufacturer´s instructions. The quantification of DNA and RNA were fluorimetrically performed. Qubit dsDNA high-sensitivity assay kit (Invitrogen, Carlsbad, CA, USA) was used to quantify DNA and Qubit RNA high-sensitivity assay kit (Invitrogen) was used to quantify RNA.

For NGS, we used the Oncomine Comprehensive Assay v3 (OCAv3, Thermo Fisher Scientific, Waltham, MA, USA). OCAv3 is a pancancer targeted NGS panel screening for 161 genes, detecting hotspot mutations in 87 genes, covering all exons of 48 genes, detecting copy number variations (CNVs) in 47 genes, and detecting 51 gene fusions (inter- and intragenic). An Ion Chef Instrument (Thermo Fisher Scientific, Waltham, MA, USA) was used for the automated libraries preparations. NGS libraries were sequenced in an Ion S5 using Ion 540 Chef Kit (Thermo Fisher Scientific) and the data were analyzed using Ion Reporter Software 5.10 with default settings (Thermo Fisher Scientific). DNA and RNA sequencing data were accessed through the Torrent Suite program (5.10 version-Thermo Fisher Scientific). Reads were aligned with the GRCh37-hg19 human reference genome, and potential mutations and copy number alteration were called with the use of Ion Reporter™ Software (5.10 version-Thermo Fisher Scientific), these processes were done following the manufacturer´s instructions (https://www.thermofisher.com/es/en/home/technical-resources/technical-reference-library/next-generation-sequencing-support-center/ngs-software-support.html). These processes were done following the manufacturer´s instructions. Additionally, we incorporated an analysis filter selecting only those variants with >500 reads and allelic frequency >5% [43]. The variant calls were manually checked using the integrative genomics viewer (IGV, Broad Institute, Cambridge, MA, USA). Germline mutations were considered based on allelic frequency close to 50% and using the Exome aggregation consortium database (http://exac.broadinstitute.org/) and the human genetic variation database (http://www.genome.med.kyoto-u.ac.jp/ SnpDB) DNA mutations and CNVs were analyzed in 40 tumors, whereas gene fusions were only tested in 35 cases. *ARID1A* was excluded from our analysis because our NGS technique has poor specificity to discriminate nucleotide changes in homopolymer regions and *ARID1A* tends to accumulate mutations in these regions [44]. Fusions detected by NGS were confirmed by RT-PCR and Sanger sequencing. The primers used for target amplification are presented in Table S5. Genes differentially mutated between glandular and solid areas were reviewed to avoid false negative results in any area due to low coverage. Figure 1 showed a combination of the mutations in solid and glandular compartments.

### 4.4. MLH1 Promoter Methylation

All cases with loss of MLH1 expression were tested for *MLH1* promoter methylation to discriminate sporadic tumors from presumptive Lynch syndrome associated carcinomas using the EpiTect Fast DNA Bisulfite Kit and EpiTect MSP Kit (Qiagen GmbH, Hilden, Germany) following the manufacturer’s instructions. DNA was extracted from complete tissue sections. No attempt was made to separately analyze glandular and solid areas.

### 4.5. Fluorescence In Situ Hybridization

Detection of translocations involving human *NTRK1* region (1q23.1) was performed by FISH analysis using the ZytoLight SPEC NTRK1 Dual Color Break Apart Probe (ZytoVision GmbH, Bremerhaven, Germany). Cutoff value was set at 15%. *CCND1* amplification status was analyzed using ZytoLight SPEC CCND1/CEN 11 Dual Color Probe (ZytoVision GmbH, Bremerhaven, Germany). A CCND1/CEN 11 ratio >2.0 was considered positive. Study of chromosomal region 8q24.21 harboring the *MYC* gene was carried out with ZytoLight SPEC MYC Dual Color Break Apart Probe (ZytoVision GmbH, Bremerhaven, Germany).

### 4.6. Public Repository Data Analysis

In order to compare our molecular results with other studies that included a more extensive cohort of HG-CRC, mutations and clinical data of Giannakis et al. 2016 [14] were downloaded from cBioPortal. Due to the mutation dataset was generated through WES, we subset 114 genes of our gene panel included in our analysis. The resulting mutation dataset was divided into two groups for study differences in the mutation profiles: Differentiation Grade (Well-Moderate and Poorly) and MSI Status (MSI-High and MSS). We performed statistical analysis using maftool package in R and an exact Fisher-test to find overrepresented mutated genes and frequency comparison.

### 4.7. Statistical Analysis

Contingency tables were used to summarize the relationship between categorical variables, such as sex, T (TNM 8th edition) stage and protein expression according to IHC staining. TNM stage was divided into two categories (I & II and III & IV) for analysis. T-test (with Welch’s correction when with unequal variance) was applied for analysis of age and to compare CD8+ tumor infiltrating cells (TILs) in the epithelium between MMRd and MMRp CRCs. Overall survival was plotted in a Kaplan–Meier curve. A long-rank test and a univariate Cox proportional hazard model was used to compare this parameter. Multivariate Cox proportional hazard regression model was also used to evaluate the prognostic significance of the variables. The data were analyzed using statistical software IBM SPSS V19 (Armonk, NY, USA). Differences were considered significant with *p* values <0.05.

## 5. Conclusions

In summary, this study shows that intertumor and intratumor molecular heterogeneity in HG-CRCs is mainly due to MMR status.

## Figures and Tables

**Table 1:** Clinicopathological differences between MMRp and MMRd CRCs.

Variables		MMRp	MMRd	All	*p* Value
	*n*	11 (27.5%)	29 (72.5%)	40	
	Age (mean)	74.64 ± 7.553	69.79 ± 14.644	71.12 ±13.168	0.181
Sex	Female	3 (27.3%)	15 (51.7%)	18	0.165
	Male	8 (72.7%)	14 (48.3%)	22	
Localization	Right colon	8 (72.7%)	22 (75.9%)	30	0.838
	Left colon & rectum	3 (27.3%)	7 (24.1%)	10	
TNM stage	I & II	0 (0.0%)	9 (31.0%)	9	0.036
	III & IV	11 (100.0%)	20 (69.0%)	31	
Protein expression	CK20 ≥ 10%	9 (81.8%)	16 (55.2%)	25	0.120
	CDX2 ≥ 10%	7 (63.6%)	14 (48.3%)	21	0.385
	p53 mutant pattern	5 (45.5%)	1 (3.4%)	6	<0.001
	Loss of ARID1A	0 (0.0%)	12 (41.4%)	12	0.011
	MET ≥ 10%	1 (9.1%)	16 (55.2%)	17	0.008
	PDL1 > 1%	2 (18.2%)	17 (58.6%)	19	0.022
	CD8 (mean)	2.27 (±3.379)	10.38 ± 8.533	8.15 ± 8.285	<0.001

Continuous variables are expressed as mean ± standard deviation. Percentages are given for discrete variables.

## Data Availability

The data presented in this study are available on Supplementary Files.

## Supplementary Materials

The following are available online at https://www.mdpi.com/2072-6694/13/2/233/s1, Figure S1: Oncoplot summarizing the molecular alterations found in Giannakis’ series applying the same panel of genes described in our series, Figure S2: Oncoplot summarizing the molecular alterations found in high-grade tumors in Giannakis’ series applying the same panel of genes described in our Figure 1, Figure S3: Most significant different genes mutated between well-differentiated and undifferentiated microsatellite stable tumors in Giannakis’ series, Figure S4: Most significant different genes mutated between well-differentiated and undifferentiated microsatellite unstable tumors in Giannakis’ series, Figure S5: Evolutionary trees of all high-grade colorectal carcinomas included in our series, Figure S6: Representative positive and negative figures of immunohistochemical staining, Table S1: Microsatellite instability analyzed by patient, Table S2: Clinicopathological data by patient, Table S3: Complete set of mutations detected in glandular and solid areas, Table S4: Immunohistochemistry antibodies, Table S5: Primers designed for the validation of fusions and their location in the target, Table S6: Differences between MSI and MSS CRCs according to stage in Giannakis’ series.
